# Osteotomy and Autograft Lengthening for Intra-Articular Malunion of the Proximal Ulna: A Case Report

**DOI:** 10.1155/2009/647126

**Published:** 2009-12-15

**Authors:** Job N. Doornberg, René K. Marti

**Affiliations:** Academic Medical Center Amsterdam, Orthotrauma Research Center Amsterdam, University of Amsterdam, Meibergdreef 9, 1100 DD Amsterdam Z-O, The Netherlands

## Abstract

An osteotomy with interposition of iliac crest bone graft and lengthening of the proximal ulna can be used to restore ulnohumeral congruency after a malunited comminuted olecranon fracture treated with figure-of-eight tension band wiring.

## 1. Introduction

Nondisplaced fractures of the olecranon process of the ulna can be treated conservatively. Figure-of-eight tension band wiring is solely indicated for dislocated transverse intra-articular fractures of the olecranon. Comminuted fractures of the olecranon are best treated with open reduction and plate fixation [1–5].

We report on a patient with an isolated severe comminuted intra-articular fracture of the olecranon originally treated with tension band wiring. She subsequently had ulnar shortening, depression of the articular surface, and flexion malalignment of the olecranon tip with 90 degrees of flexion and a 25 degree flexion contracture. A reconstructive procedure consisting of lengthening of the olecranon with autograft interposition in conjunction with an osteotomy to correct flexion and depression and stable plate fixation led to a functional result. 

## 2. Case Report

**Figure 1:**
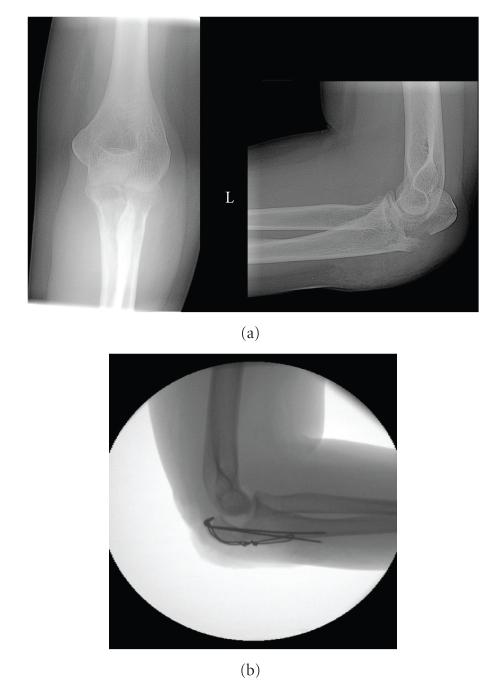
A 24-year-old previously healthy woman fell from standing height directly on her left elbow and sustained an isolated comminuted olecranon fracture (a) She was treated in an outside hospital with a figure-of-eight tension band construct. Intraoperative views with an image intensifier reveal marginal reduction of the fragments and incongruency of the ulnohumeral joint (b).

A 24-year-old previously healthy woman fell from standing height directly on her left elbow and sustained an isolated comminuted olecranon fracture with axial impaction and depression of the articular joint surface (Figure 1(a)). The radial head was intact. She was treated in an outside hospital with a figure-of-eight tension band construct despite the presence of intraarticular comminution. Intraoperative views with an image intensifier reveal marginal reduction of the fragments and incongruency of the ulnohumeral joint (Figure 1(b)). She was immobilized in a cast for 10 days and was not allowed passive nor active range of motion exercises. Ten days after the injury the patient was evaluated for routine postoperative follow-up. Radiographic evaluation at that time revealed ulnar shortening and poor ulnohumeral congruency. After 13 weeks she underwent a second procedure for removal of prominent hardware.

**Figure 2:**
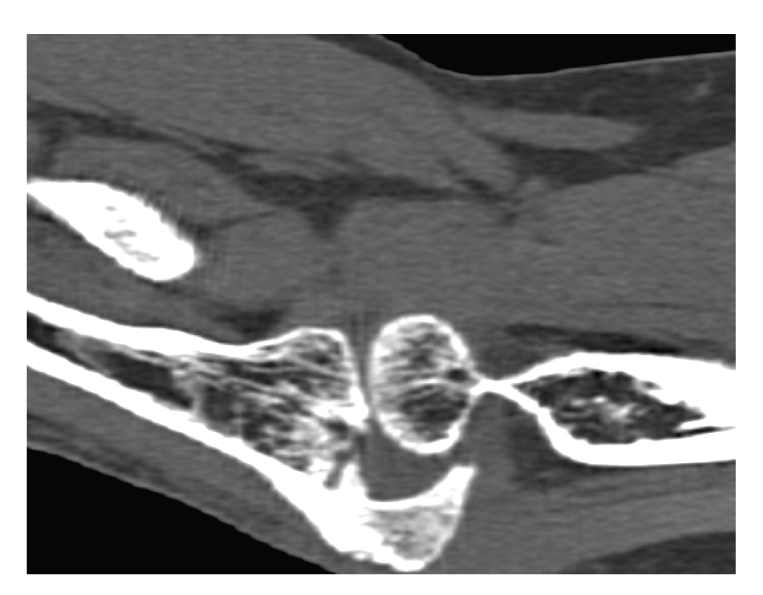
Computed tomographic evaluation revealed depression of the articular surface of the trochlear notch and shortening of the proximal ulna. She had an incongruent ulnohumeral joint leading to impingement and loss of function. The articular surfaces of the radio-capitellar joint remained opposed.

Five months after the injury, the patient presented to the senior author with a poor functional result. She had 65 degrees of ulnohumeral motion with 90 degrees of flexion and a 25 flexion contracture with full forearm rotation. She had a stable elbow with pain in active and passive range of motion. Computed tomographic evaluation revealed depression of the articular surface of the trochlear notch and shortening of the proximal ulna. She had an incongruent ulnohumeral joint leading to impingement and loss of function. The articular surfaces of the radiocapitellar joint remained opposed (Figure 2). 

**Figure 3:**
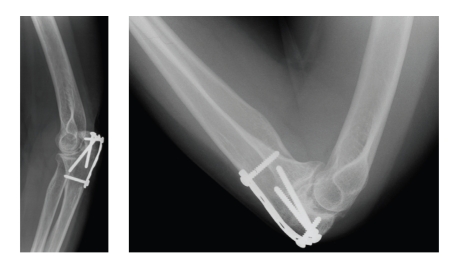
An iliac-crest bone graft, 6 mm by 15 mm in size, was interposed to widen and reconstruct the trochlear notch. After lengthening of the proximal olecranon, the humerus could be easily reduced to restore ulnohumeral congruency. Stable fixation was achieved with 2 lag screws secured with a 4-hole LCP plate.

An osteotomy was planned to restore congruency of the trochlear notch to achieve a functional arc of motion. After induction of general anesthesia, the patient was placed in lateral decubitus and the left arm was placed over a bolster. A midline posterior incision was used to expose the proximal ulna. An osteotomy was performed at the level of the depressed articular surface to mobilize and elevate the depressed fragments in an attempt to realign the articular surfaces of the ulnohumeral joint. Reduction of the malunited intra-articular fragments was not possible because the fracture fragments were sclerotic. Shortening of the olecranon due to axial impaction—which was worsened by the tension-band construct—prohibited good alignment. An iliac-crest bone graft, 6 mm by 15 mm in size, was interposed to widen and reconstruct the trochlear notch. After lengthening of the proximal olecranon, the humerus could be easily reduced to restore ulnohumeral congruency. Stable fixation was achieved with 2 lag screws secured with a 4-hole LCP plate (Figure 3). It was noted that the posterior capsule was contracted. However, at this point it was chosen not to perform a posterior capsular release to preserve the surrounding soft tissues of the olecranon tip to protect vascular blood supply. Postoperatively, the patient was immobilized in a long arm cast and continuous passive motion was initiated 24 hours after surgery and therapy was started. Careful active motion also began immediately under the supervision of a physical therapist.

One year after the index surgery, the patient had a functional arc of motion. She had 95 degrees of ulnohumeral motion with 115 degrees of flexion and a flexion contracture of 20 degrees. Her elbow was rendered stable and she has no pain. Posterior contracture release was performed at this point combined with hardware removal. Postoperative radiographs suggested posterior impingement of the olecranon tip hindering extension. This was not found on Intraoperative passive range of motion.

**Figure 4:**
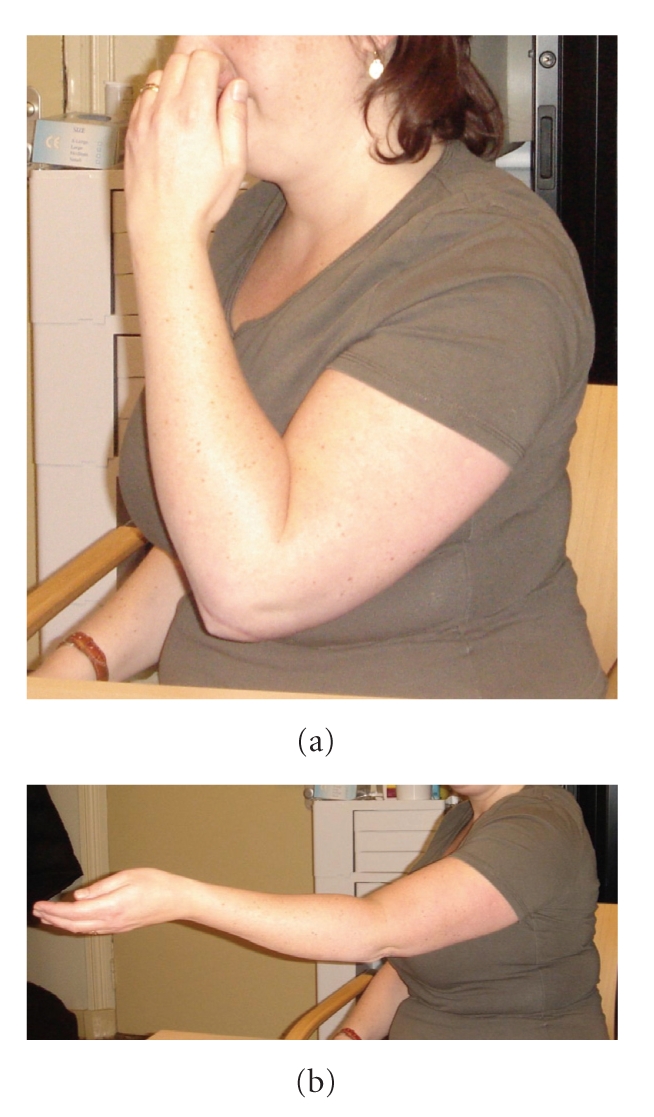
Two years after the index surgery she had a functional elbow with 105 degrees of ulnohumeral motion (115 degrees of flexion (a) and a 10-degree flexion contracture (b)), 87 points according to American Shoulder and Elbow Surgeon evaluation and 7 points on the Disability of Arm Shoulder and Hand score.

Two years after the index surgery, and one year after removal of hardware and contracture release, she had a functional elbow with 105 degrees of ulnohumeral motion (115 degrees of flexion and a 10-degree flexion contracture), 87 points according to American Shoulder and Elbow Surgeon evaluation and 7 points on the Disability of Arm Shoulder and Hand score (see Figure 4).

## 3. Discussion

This is the first report in literature of an olecranon osteotomy with autograft lengthening to widen and reconstruct the trochlear notch and thus restoring ulnohumeral alignment after olecranon malunion. It is well established that effective treatment of fracture-dislocations of the olecranon requires a stable trochlear notch [3, 6]. Simple transverse fractures only benefit from figure-of-eight tension band wiring with predictable outcomes [1, 7, 8]. The goal of this technique is to convert the extensor force of the triceps to a dynamic compression force along the articular surface [9–11]. This compressive force results in shortening of the trochlear notch if the fracture is comminuted as is illustrated in this case. 

Postoperative immobilization further impaired a functional result in this case. Internal fixation must restore articular congruency and must be stable enough to allow for early mobilization. In this case the patient was immobilized for 10 days without continuous passive motion directly postoperative which resulted in only 65 degrees of ulnohumeral motion. However, impingement due to the depressed articular surface was the main reason for dysfunctional range of motion.

## 4. Conclusion

An osteotomy with interposition of iliac crest bone graft and lengthening of the proximal olecranon can be used to restore the trochlear notch and ulnohumeral congruency. Although technically challenging, a functional arc of motion can be achieved in the young and active patient.
